# Feeding the Infant at High-Risk of Celiac Disease – An Update

**DOI:** 10.3389/fped.2015.00047

**Published:** 2015-05-26

**Authors:** Robert N. Lopez, Andrew S. Day

**Affiliations:** ^1^Department of Paediatrics, University of Otago Christchurch, Christchurch, New Zealand

**Keywords:** children infants, celiac disease, HLA genes, breast feeding, gluten

Celiac disease is a life-long condition with significant morbidity and health-care costs (1). Conventional understanding of etiopathological factors, specifically the roles played by the HLA genes (DQ2 and DQ8), has greatly advanced in recent years. Furthermore, it is now well accepted that these HLA genes contribute to an increased risk of celiac disease for first-degree relatives of those affected by the condition. One such group is infants born to a mother with known celiac disease. Current guidelines for such high-risk infants include the recommendation to introduce gluten between 4 and 6 months of age and ideally, while still breastfeeding. Emerging data from a number of large studies refute the basis for these recommendations, thereby necessitating a new approach.

Existing guidelines pertaining to gluten introduction are largely based on observational, retrospective data. In 1980s, the Swedish epidemic of celiac disease occurred following a general recommendation to delay the introduction of gluten to beyond 6 months of age (2). The dramatic increase in celiac diagnoses was halted in Sweden when this recommendation was revised so that gluten was instead introduced at 4 months of age. Subsequent evidence emerged showing that the introduction of gluten, either prior to 3 months or after 7 months of age, increased the risk of celiac disease (3).

Similarly, the advice to introduce gluten while infants were still being breastfed was in keeping with the best available evidence, which suggested a negative association between duration of breastfeeding and the development of celiac disease. A series of large prospective studies conducted in recent years has now provided new evidence on which to base our management of these infants at greater risk of celiac disease.

In a well-designed study conducted across multiple locations in Italy, Lionetti et al. (4) confirmed that children with a high-risk HLA genotype (DQ2 or DQ8 positive) were more likely than those with standard risk HLA genotype (DQ2 or DQ8 negative) to develop celiac disease autoimmunity. Interestingly, following randomization to having gluten introduced at either 6 or 12 months of age, there was no difference in the rates of celiac disease autoimmunity or biopsy-proven celiac disease between the groups. Results from this study did, however, demonstrate that delayed introduction of gluten was associated with a later onset of disease. In this study, breastfeeding had no modifying effect on subsequent development of celiac disease.

A further multicentre, double-blinded randomized controlled trial included children who were either HLA-DQ2 or HLA-DQ8 positive and who had a first-degree relative with celiac disease (5). Each arm was randomized to receive either a small amount of gluten or placebo at between 4 and 6 months of age. This study reported no difference in the incidence of celiac disease between the groups at 3 years of age. The authors’ also conclude that exclusivity or duration of breastfeeding appeared to have no impact on celiac disease development in this cohort.

From the Netherlands, a nested subset of children who were either HLA-DQ2 or HLA-DQ8 positive within a population-based cohort study were retrospectively assessed with regards to gluten introduction, breastfeeding, and the development of celiac disease (6). The findings arising from this study demonstrated that neither the delayed introduction of gluten to beyond 6 months of age nor duration of breastfeeding of at least 6 months had any effect on developing celiac disease autoimmunity. Similar to the Dutch study, a genetically high-risk sub-group within a multi-national birth cohort was followed up with regards to risk factors for subsequent development of celiac disease (7). In the latter study, gluten introduction either before 4 months or after 6 months of age appeared to have no effect on celiac disease risk.

By contrast, evidence from a Norwegian cohort study suggested that introduction of gluten after 6 months of age and breastfeeding beyond 12 months of age conferred additional risk for developing celiac disease (8). It is important to note, however, that the cohort in this Norwegian study was not stratified according to genetic risk (unlike the previously referenced reports).

While each of the recent studies has provided useful evidence, it is important to note that these reports have done so by asking slightly different questions. The consensus, however, is clear – the approach to a first-degree infant of someone with celiac disease must change. Although it still appears appropriate and important to introduce gluten-containing foods in mid-infancy, the previous recommendation of coordinating this with ongoing breastfeeding is no longer valid. That said, the myriad benefits of breastfeeding still hold true: none of the presented evidence warrants changing advice in this regard. While several aspects of the optimal management of genetically susceptible infants are not known, these recent studies at least necessitate revision of the current recommendations.

## Conflict of Interest Statement

The authors declare that the research was conducted in the absence of any commercial or financial relationships that could be construed as a potential conflict of interest.
